# Vesicular Nucleo-Cytoplasmic Transport—Herpesviruses as Pioneers in Cell Biology

**DOI:** 10.3390/v8100266

**Published:** 2016-09-27

**Authors:** Thomas C. Mettenleiter

**Affiliations:** Institute of Molecular Virology and Cell Biology, Friedrich-Loeffler-Institut, 17493 Greifswald-Insel Riems, Germany; thomas.mettenleiter@fli.de; Tel.: +49-383-517-1250

**Keywords:** herpesvirus, nuclear egress, nuclear egress complex, nucleo-cytoplasmic transport

## Abstract

Herpesviruses use a vesicle-mediated transfer of intranuclearly assembled nucleocapsids through the nuclear envelope (NE) for final maturation in the cytoplasm. The molecular basis for this novel vesicular nucleo-cytoplasmic transport is beginning to be elucidated in detail. The heterodimeric viral nuclear egress complex (NEC), conserved within the classical herpesviruses, mediates vesicle formation from the inner nuclear membrane (INM) by polymerization into a hexagonal lattice followed by fusion of the vesicle membrane with the outer nuclear membrane (ONM). Mechanisms of capsid inclusion as well as vesicle-membrane fusion, however, are largely unclear. Interestingly, a similar transport mechanism through the NE has been demonstrated in nuclear export of large ribonucleoprotein complexes during *Drosophila* neuromuscular junction formation, indicating a widespread presence of a novel concept of cellular nucleo-cytoplasmic transport.

Whereas intracytoplasmic vesicular transport is well established, nucleo-cytoplasmic transport has so far been thought to be restricted to passage through the nuclear pore either passively, if size permits, or via karyopherin-mediated active transport. This limits transport in and out of the nucleus to particles of a maximum of ca. 39 nm (reviewed in [1]). With a diameter of ca. 120 nm herpesvirus capsids, which are assembled in the nucleus but mature to infectious virions in the cytosol, are unable to pass through the nuclear pore. It has become clear in the last decade that they leave the nucleus and traverse the nuclear envelope (NE) by a vesicle-mediated process that entails budding of nucleocapsids at the inner nuclear membrane (INM), thereby forming a primary enveloped virion in the perinuclear space. The primary envelope then fuses with the outer nuclear membrane (ONM) (recently reviewed in [2,3]). Long thought to be specific for herpesviruses, this pathway has recently also been suggested to function in the export of large ribonucleoprotein (RNP) complexes during development of *Drosophila* [4,5]. Common between the two is the involvement of kinases (viral, cellular, or both) to phosphorylate and soften the nuclear lamina allowing access of the ‘cargo’ (i.e., viral nucleocapsids or cellular RNPs) to the INM as well as morphological similarities [6]. The cellular AAA+ ATPase TorsinA has also been proposed to be involved in both processes [7,8]. Thus, the notion was developed that herpesviruses have actually co-opted a hitherto cryptic cellular transport pathway for their replication.

Although our current understanding of nuclear egress is mostly based on static imaging data lacking information on the dynamics of the process, recently, molecular details on this novel nucleo-cytoplasmic transport have been elucidated in the herpesviruses [9,10]. Two proteins that are conserved in sequence between members of the family *Herpesviridae* (i.e., the ‘classical’ herpesviruses) within the order *Herpesvirales* form a heterodimeric nuclear egress complex (NEC). The NEC exhibits a highly conserved structure similar in shape and size in the alphaherpesviruses herpes simplex virus 1 (HSV-1) [11] and pseudorabies virus (PrV) [11,12], and the betaherpesvirus human cytomegalovirus (HCMV) [13,14] (Figure 1). The C-terminally membrane-bound component (designated pUL34 in the alphaherpesvirusesHSV-1 and PrV) exhibits a large groove in the globular head domain into which an N-terminally extended α-helix of the otherwise globular pUL31 integrates. A bent α-helix at the end ‘locks’ the interaction in place. The pUL31 structure is maintained by an intramolecular zinc finger motif coordinated by four conserved residues, three cysteines and a histidine. The NEC alone is sufficient for formation of vesicles from the INM even in the absence of nucleocapsids [15,16] as well as for membrane bending and scission in artificial simple membrane systems (i.e., giant unilamellar vesicles (GUVs)) [17,18]. While pUL34 provides anchorage to and probably maintains an appropriate distance of pUL31 from the INM for formation of uniformly sized vesicles [10,15], pUL31 alone is able to mediate vesicle formation from GUVs when artificially tethered to the membrane [18]. Thus, pUL31 seems to be the ‘business end’ of the NEC. Nuclear egress complex polymerization results in the formation of a hexagonal lattice in model and eukaryotic membranes serving as a coat underlying the membrane in the resulting vesicles [10,17]. Although not all molecular details of NEC coat formation have been elucidated, recent studies have shed some light on this process [9,12].

In contrast, little information is available on the molecular mechanism of cargo (i.e., nucleocapsid) incorporation or fusion between the vesicle membrane and the ONM. Incorporation of nucleocapsids into nascent primary envelopes requires interaction between capsid-associated proteins and the NEC. Evidence for the binding of either component of the NEC to nucleocapsids has been published [19,20,21] and is thought to occur via the heterodimeric capsid vertex specific complex (CVSC) consisting of pUL17 and pUL25 [22]. However, it remains unclear how the NEC mediates nucleocapsid incorporation and which interaction interfaces are involved. Recently, a refined structure of the intranuclear HSV-1 nucleocapsid has been obtained [23], which indicates that, besides pUL25 within the CVSC, the large tegument protein pUL36 may also decorate intranuclear capsids at the penton sites, thus establishing the possibility that this protein may also be involved in capsid docking during nuclear egress. With the availability of the NEC structure it is now possible to identify potential interacting domains that, most likely, reside exclusively within the exposed part of pUL31. Non-structured regions at the membrane-distal face of pUL34-bound pUL31, which are flanked by conserved alpha-helices, are prime candidates for this role (see Figure 1, red circles).

Fusion between the primary envelope and the ONM, a prerequisite for nucleocapsid release into the cytosol, remains enigmatic. The fusion machinery conserved within the *Herpesviridae* family consists of the core fusogen gB and the heterodimeric gH/gL complex [24]. While both components are essential for infectious entry, they are apparently not required for nuclear egress [25,26]. Absence of the alphaherpesviral kinase pUS3 significantly delays but does not block nuclear egress (reviewed in [2]). Thus, other fusion mediators have to be involved. Recent studies indicated that cellular proteins may actually mediate this particular fusion event. Components of the cellular endosomal sorting complexes required for transport (e.g., ESCRT-III) are involved in formation and scission of cytoplasmic vesicles including formation of multivesicular bodies (reviewed in [27]), which is topologically similar to primary envelopment. However, they have also been implicated in nuclear pore complex assembly [28] and NE repair processes [29,30,31] as well as in nuclear egress of the gammaherpesvirus Epstein-Barr virus (EBV) by interacting with the pUL31 homolog BFRF1 [32] and thus may play a role in de-envelopment. AAA+ ATPases may also be involved such as TorsinA [7,8] or its regulator LULL1 [33]. Fusion processes including the nuclear membranes also occur during nuclear pore assembly [34,35], where nucleoporins POM121 and Nup107-160 are instrumental in INM/ONM fusion [34]. Whether these pathways are indeed involved in herpesvirus nuclear egress is unclear.

Host cell p32, cluster of differentiation (CD) 98 heavy chain, and β1 integrin have been implicated in fusion during nuclear egress as well [36,37]. The linker of nucleoskeleton and cytoskeleton (LINC) complex may also be modulated during nuclear egress since it restricts the width of the perinuclear space (PNS) to ca. 40 nm and needs to be dissolved and/or displaced for accommodation of the ca. 120 nm nucleocapsid. One of its components, Sun2, is degraded during HCMV infection [38], demonstrating that it is a target during herpesvirus infection. Moreover, cells expressing a dominant-negative form of soluble luminal Sun2 exhibit impaired nuclear egress of PrV [39]. However, so far no viral or cellular protein has been convincingly shown to be crucial for mediating the fusion between primary envelope and ONM.

While the NEC structure of different members of the ‘classical’ herpesviruses has now been solved and shown to be highly similar reflecting significant sequence conservation, no sequence homologs of the NEC components of the *Herpesviridae* family have been found in herpesviruses of fish and amphibia (family *Alloherpesviridae*) or molluscs (family *Malacoherpesviridae*) [40,41]. However, ultrastructurally, no gross differences in the viral replication cycle between the alphaherpesvirus PrV and the alloherpesvirus koi herpesvirus (KHV) cyprinid herpesvirus 3 (CyHV-3) were observed. Nuclear egress appears to occur along a similar, if not identical, pathway [42]. Thus, alloherpesviruses most likely encode an NEC whose components do not exhibit overt sequence homology with the NEC of the classical herpesviruses. To understand how nuclear egress is mediated by herpesviral proteins unrelated in sequence but presumably related in structure, identification and characterization of the NEC of an alloherpesvirus may provide evidence on what putative cellular counterparts of the NEC may look like.

Besides herpesviruses, other viruses which assemble in the nucleus have to cross the NE (reviewed in [1]). Although parvoviruses at diameters of 18–26 nm are small enough to pass through the nuclear pore, they perforate the NE for early release. Adenoviruses and polyomaviruses also alter NE integrity for capsid release. In contrast, the nuclear egress of baculoviruses and nucleorhabdoviruses resembles the mechanism found in herpesviruses (i.e., primary envelopment at the INM to form an intraluminal primary virion) [43,44,45]. However, the molecular basis for this primary envelopment and the subsequent steps of nucleocapsid translocation are completely unclear. In the context of the gain of knowledge in herpesviruses and the ‘gap’ between herpesvirus vesicular nucleo-cytoplasmic transport and that of large RNP complexes in *Drosophila*, the study of nuclear egress mechanisms of these viruses may assist in acquisition of a more general picture of this novel intracellular transport pathway.

Vesicular transport through the NE could serve several purposes in normal cellular metabolism. It could transport functional large RNP complexes nuclear pore-independently through the NE for the coordinated translation of mRNAs as shown for *Drosophila* neuromuscular junction formation [4]. However, it may also transfer misfolded nuclear protein aggregates to the cytosol for degradation [46]. This vesicular transfer would be particularly relevant in cells in which NE breakdown does not occur (i.e., terminally differentiated G_0_ cells) and no alternative for large cargo to cross the NE barrier exists. Thus, there are good biological reasons for the presence of this pathway. However, conclusive evidence that it represents a general nucleo-cytoplasmic transport mechanism that is present in many, if not all, cells has yet to be obtained.

## Figures and Tables

**Figure 1:**
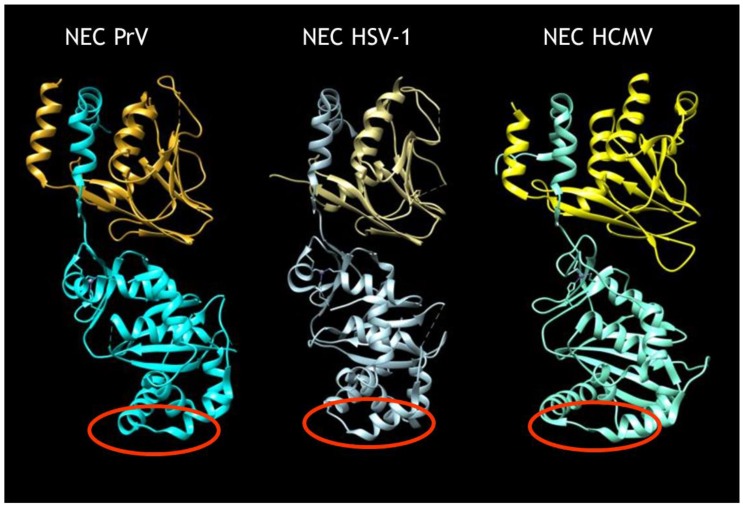
Structure of herpesvirus nuclear egress complex (NEC). Structures of the heterodimeric NEC of the alphaherpesviruses pseudorabies virus (PrV) [11,12] and herpes simplex virus 1 (HSV-1) [11] as well as of the betaherpesvirus human cytomegalovirus (HCMV) [13,14]. The N-terminal extended α-helix of pUL31 (**bottom**) inserted into the groove in pUL34 (**top**) is apparent. In the HSV-1 NEC structure, the second pUL34 α-helix lining the groove has not been resolved. Red circles denote putative capsid-interaction domains.
